# Effects of using structured templates for recalling chemistry experiments

**DOI:** 10.1186/s13321-016-0118-6

**Published:** 2016-02-19

**Authors:** Cerys Willoughby, Thomas A. Logothetis, Jeremy G. Frey

**Affiliations:** Faculty of Natural and Environmental Sciences, University of Southampton, Southampton, SO17 1BJ UK

**Keywords:** Templates, Experiments, Experiment record, Context, ELN, User experience, Study

## Abstract

**Background:**

The way that we recall information is dependent upon both the knowledge in our memories and the conditions under which we recall the information. Electronic Laboratory Notebooks can provide a structured interface for the capture of experiment records through the use of forms and templates. These templates can be useful by providing cues to help researchers to remember to record particular aspects of their experiment, but they may also constrain the information that is recorded by encouraging them to record only what is asked for. It is therefore unknown whether using structured templates for capturing experiment records will have positive or negative effects on the quality and usefulness of the records for assessment and future use. In this paper we report on the results of a set of studies investigating the effects of different template designs on the recording of experiments by undergraduate students and academic researchers.

**Results:**

The results indicate that using structured templates to write up experiments does make a significant difference to the information that is recalled and recorded. These differences have both positive and negative effects, with templates prompting the capture of specific information that is otherwise forgotten, but also apparently losing some of the personal elements of the experiment experience such as observations and explanations. Other unexpected effects were seen with templates that can change the information that is captured, but also interfere with the way an experiment is conducted.

**Conclusions:**

Our results showed that using structured templates can improve the completeness of the experiment context information captured but can also cause a loss of personal elements of the experiment experience when compared with allowing the researcher to structure their own record. The results suggest that interfaces for recording information about chemistry experiments, whether paper-based questionnaires or templates in Electronic Laboratory Notebooks, can be an effective way to improve the quality of experiment write-ups, but that care needs to be taken to ensure that the correct cues are provided.

**Graphical abstract:**
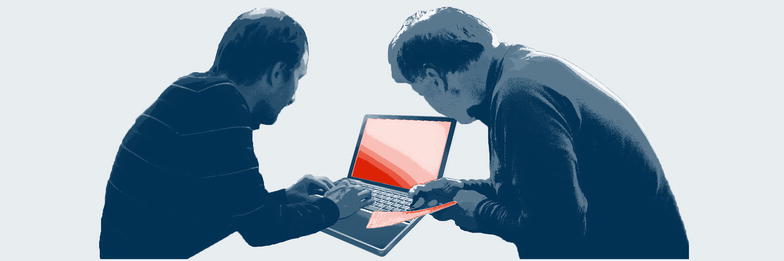
Scientists have traditionally recorded their research in paper notebooks, a format that provides great flexibility for capturing information. In contrast, Electronic Laboratory Notebooks frequently make use of forms or structured templates for capturing experiment records. Structured templates can provide cues that can improve record quality by increasing the amount of information captured and encouraging consistency. However, using the wrong cues can lead to a loss of personal elements of the experiment experience and frustrate users. This image shows two participants from one of our studies recording their experiment using a computer-based template

**Electronic supplementary material:**

The online version of this article (doi:10.1186/s13321-016-0118-6) contains supplementary material, which is available to authorized users.

## Background

For a scientist, the structure and information contained in the scribbles in our notebooks are likely to be a combination of the result of classroom learning, socialisation to professional practice, and our own personal style [1]. When it comes to entering information on a computer, however, what we record is likely to be influenced by the design of the interfaces we are using. In particular, the questions we are asked and the prompts that we are given will change what information we provide. Electronic Laboratory Notebooks (ELNs) frequently make use of forms or structured templates to capture information. What is not known is whether providing a formal structure for the recording of experiments will be beneficial or if they may present negative consequences by potentially constraining or changing the information that is recorded.

The keeping of good records is essential in laboratory science, to capture both the thoughts of the experimenter and the detailed procedures of the experiments [2]. It is important that records of scientific activities are accurate, complete, and accessible. If the record is missing information, contains incorrect data, or is difficult to understand, then it cannot serve its function as a tool of learning and support for the scientist to whom it belongs, to members of the research group who may need to make sense of it, and a broader audience if the ownership or reputation of the research is at stake. In both education and research settings, capturing the experiment record is more than just capturing the experiment procedure. In addition to recording ‘What did I do?’ information, the record must capture quality information about the experiment design, the materials used and produced including sample or batch numbers, what was observed, explanations for actions taken or unexpected results, decisions that were made, problems that occurred, how observations and events relate to the experimenter’s knowledge of chemistry, and an interpretation and evaluation of the results of the experiment. The information captured must be clear and complete enough that someone else without prior knowledge of the experiment could reproduce the experiment and understand how their new results compare to the original.

If an experiment record does not meet these quality criteria, then the full context will not available for future reference, either for the benefit of the researcher themselves, for other researchers, and for assessment of understanding and learning by the teacher or supervisor. Without this context the researcher will also struggle to justify arguments they make in their research based on the data and to critically evaluate their own performance in the experiment. The studies presented in this paper make use of these quality criteria to assess the effectiveness and impacts on quality of different methods of capturing experiment records.

The traditional medium for the capture of scientific records by students and researchers alike is the paper-notebook. While ELNs are still relatively rare in academic environments with a variety of challenges to overcome [3, 4], the advent of computers, and the digital capture of data in particular, has begun to change the way many laboratory scientists record their experiments [5]. ELNs provide a range of features that can help to improve data management, data retrieval, and collaboration providing positive benefits for teaching, learning, and research [6].

Paper notebooks are by nature blank, and offer no guidance on what information should be recorded within them. Consistency in recording comes from learning through the classroom, as part of membership of a scientific community of practice, through shared best practices, and standard guidelines for recording experiments used in both academia or formalised by regulatory organisations, for example the Good Laboratory Practice Handbook [2, 7]. Ultimately most scientists will develop their own personal style of recording by trial and error [1, 8]. Many academic researchers are still keeping their paper notebooks in exactly the same way they were when they were taught decades ago [9], and many still believe learning how to keep a paper notebook is a vital skill for students and researchers [10].

The proliferation of instruments producing data in a digital format means that even researchers still exclusively using paper notebooks have had to change their behaviour, either by printing out electronic data and pasting it into their paper-notebooks, or storing digital files and including a reference to the storage location and file names within their notebooks [9]. Some researchers have begun to record their experiments in digital format using tools including Word and Excel, or digital notebooks such as Microsoft OneNote and Evernote [11]. ELNs have the advantage of being able to automatically capture provenance and audit trail in a way that Microsoft Word or Excel files cannot [9].

ELNs can help to enable efficient and consistent recording of experiments through the use of templates that provide formal structure for data entry [12]. The vast majority of ELNs surveyed use templates or forms to enforce a standardised structure for the capture of scientific experiments.^1^

Studies in cognitive psychology have demonstrated that what we remember or choose to recall when asked is dependant upon our knowledge and previous experiences [13, 14], but other factors, in particular the use of cues, can influence and shape what is recalled [15–17]. The results of such studies have been used to design effective methods of information collection such as improving the design of surveys and questionnaires [13, 18, 19] and questioning techniques used in eye-witness interviews [20]. However, it is possible that using templates may have a negative impact. For example, other studies have indicated that the inflexibility and formality of templates might restrict the content that the author enters [21] and that important information is not recorded if the template does not specifically ask for it [22].

In this paper we discuss the results from a series of studies looking at the effect of using templates to record experiments with UG/PG students and academic researchers. The studies investigate whether using templates improves or impairs the quality of information captured by students undertaking scientific experiments. We do not believe that such a formal investigation has been applied to information capture and recall in a laboratory setting. The conditions, participants, and settings for the three studies can be viewed in Table 1.

**Table 1:** Participants and conditions for the template studies

	Participants	Conditions
Study 1	20 chemistry undergraduates	‘No Template’ and ‘Template’
Study 2	20 chemistry undergraduates	‘No Template’, ‘Titles Template’, and ‘Profile Template’
Study 3	~65 chemistry researchers and staff	‘No Template’, ‘Titles Template’, and ‘Profile Template’

## Study 1: University of Southampton Organic Chemistry Summer School (OCSS) and paper-based templates

The initial study was carried out during an annual Organic Chemistry Summer School (OCSS) run at the University of Southampton. The Summer School is run over 3 weeks after the end of term with a small group of second-year undergraduate students, enrolled on a variety of BSc and MChem programmes. The students were selected for the Summer School based on previous academic achievements. The purpose of the Summer School is to give the selected students experience of advanced lab techniques and working with industry between their second and third undergraduate year. Although it would have been preferable to use a larger group of students for the study, running the studies as part of the standard laboratory courses was not possible because of the risk of an unfair impact on work formally assessed as part of a degree. The Summer School itself does not involve any formal assessment and therefore provided an excellent opportunity to perform a study where different conditions could be assessed whilst engaging students in a realistic situation completing real chemistry experiments. Taking part in this study was a mandatory part of the Summer School. An advantage of undergraduates is that they are less likely than more experienced researchers to have developed their own personal style or bad habits.

Two experiments from the first week of the Summer School were included in this study: preparation of an Organoboronic Acid and a Radical Benzylic Bromination. Two experiments from the Summer School were used to enable each student to generate a write-up in each of two study conditions: the *No Template condition* and the *Template condition.* The students were randomly allocated to one condition for the first experiment on one day and then swapped condition for their second experiment on the second day. In each condition the students were asked to complete a paper questionnaire independently after they had completed their experiment.

The *No Template condition* was effectively a blank piece of paper, whilst the *Template condition* included a number of sections for the students to complete, each section title acting as a cue to remind the students what to record. The titles for each section were based upon the laboratory notebook and report writing guidelines given to all students within the chemistry department at Southampton University. The questionnaires used in the study and the recordkeeping guidance from the course can be found as Additional file 1: Data file 1, Additional file 2: Data file 2 and Additional file 3: Data file 3.

### Results and discussion for Study 1

The reports generated by the study were examined on a number of criteria to assess the differences between the records generated by the different conditions. The word count for the reports is used to examine whether the change in conditions result in the capture of more or less information. In order to investigate if the different conditions would have a positive or negative effect on the quality of the record and on student grades, each of the reports for both conditions were given a grade by an independent marker. The grade given was based on quality criteria important for experiment records such as the clarity and correctness of language and nomenclature, reaction scheme equation completeness and balancing, completeness and accuracy of the experiment procedure, and discussion of results, together with elements important for education such as understanding of the topic. The reports were also examined for the kinds of information that the students recorded and to identify whether they included all the information that they were expected to produce including aims, materials that they used, the actions or techniques used in the experiment, observations, explanations, and results. We consider that observations and explanations that are embedded in the correct location within the protocol description, rather than separate from it, are more valuable for experiment reproducibility, and we therefore also examine whether these elements are recorded within the experiment procedure or outside of it.

**Fig. 1:**
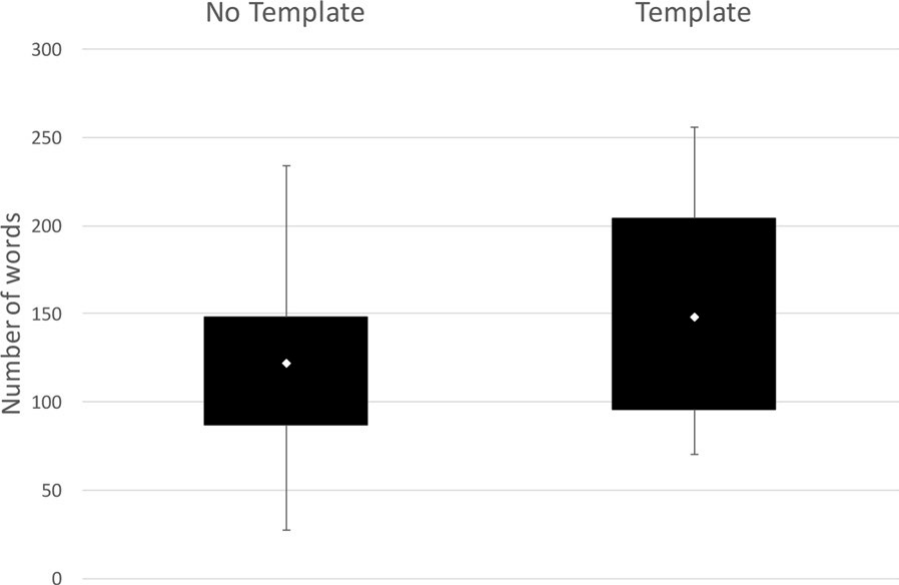
Comparison of word counts for the *No Template condition* and *Template condition* in Study 1. *Box plots* showing the range of word counts and the means for both conditions in Study 1. The figure shows that on average the *Template condition* generated more words in the reports than the *No Template condition* as we expected

As shown in Fig. 1, the average number of words used by the students to record their experiments is higher in the *Templatecondition* compared to the *No Template condition* consistent with the expectation that the *Template condition* would lead to the capture of more information. However, nearly half actually reduced their word count in the *Template condition* because the majority of these students did not complete the Discussion and Conclusion section of the template. The reason for the failure to complete these sections appears to be confusion from some students about what information they should record here, for example, a number of students recorded ‘N/A’ or ‘?’ for these sections. This confusion may be due to a lack of experience; some students indicated they felt that they couldn’t make conclusions because they did not have the complete data from their analyses.

**Fig. 2:**
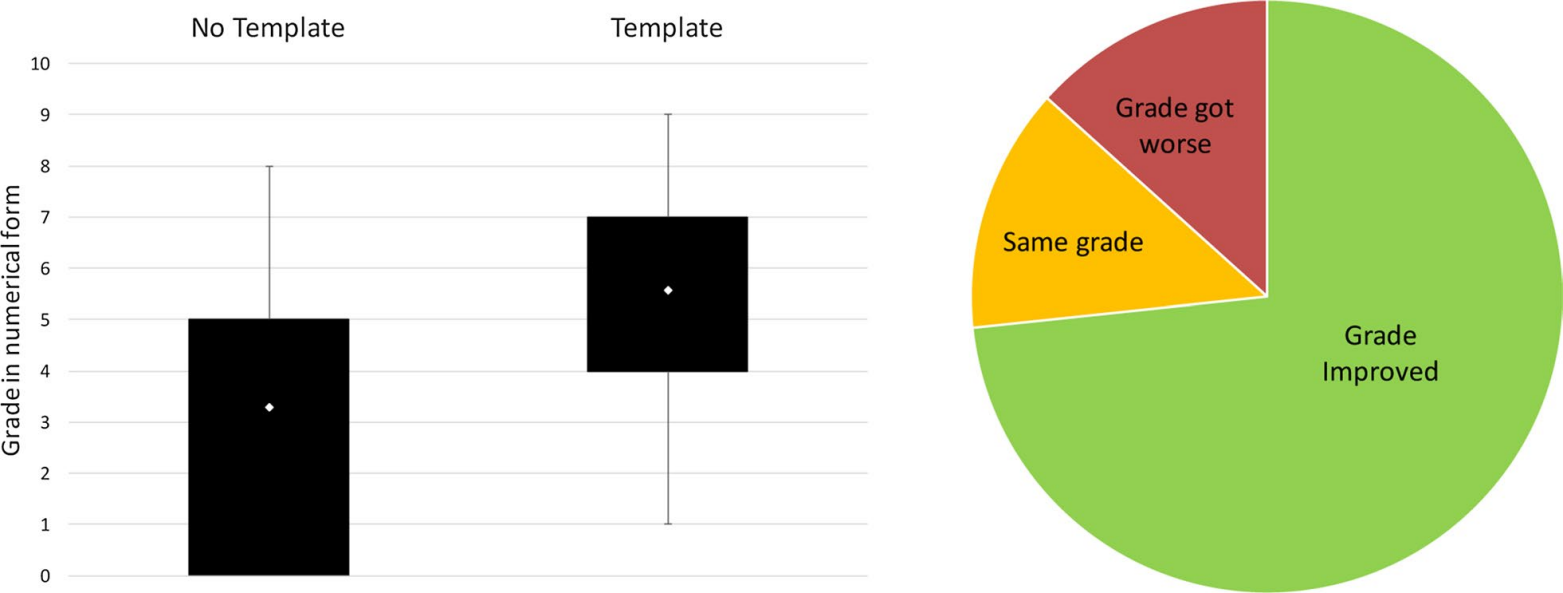
Comparison of grades for the *No Template condition* and *Template condition* in Study 1. *Box plots* showing the range of grades (converted into numerical form—where 0 is the lowest grade and 10 is the highest) and means for both conditions in Study 1, and a *Pie chart* showing how individual students grades changed with the Template. The figure shows that on average the mean and range are both higher for the *Template condition*, suggesting that the *Template condition* improved the quality of the experiment record compared to the *No Template condition*. The majority of students received an improved grade in the Template condition

Figure 2 shows the results of the independent grading exercise for Study 1, with the grades converted to a numeric value so that they can be compared across the conditions (10 represents the highest grade possible and 0 the lowest). On average the *Template condition* resulted in an increase in grade, with 55 % of those with an improved grade receiving had a grade that was significantly higher in the *Template condition* compared to their grade in the *No Template condition*. Overall this suggests that the *Template condition* resulted in an increase in the quality of the experiment record for the majority of the students.

**Fig. 3:**
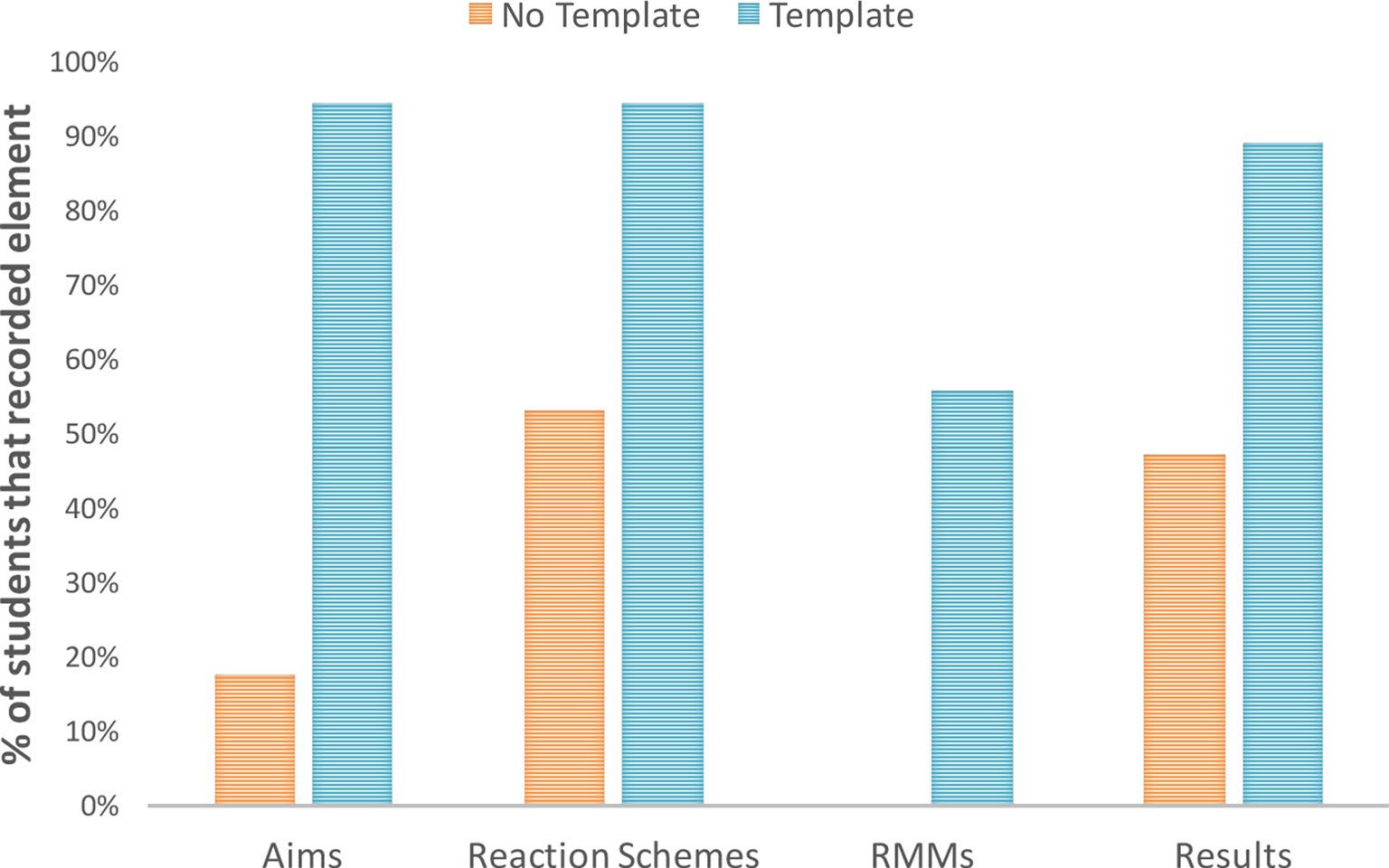
Comparison of the capture of information specifically requested in Study 1. This *bar chart* shows the percentage of students that recorded information that we specifically requested in the *Template condition* compared to the *No Template condition*: Aims, Reaction Schemes, Relative Molecular Masses, and Results information. In all cases more students recorded this information in the *Template condition* than the *No Template condition*, although it can be seen that some information, such as the results and reaction schemes were more likely to be recorded without prompting in the *No Template condition*

One of the expected effects of using the template was to encourage the recording of information requested. As can be seen in Fig. 3, the results of the first study demonstrated that this effect did occur, with more students recording the information that was specifically requested in the *Template condition* than in the *No Template condition*, particularly for the Aims and Relative Molecular Masses (RMMs) requested in the template. Not only did more students record results in the *Template condition* (90 %) compared to the *No Template* condition, but the average number of pieces of information about the results recorded is double in the *Template* condition; results recorded in the *No Template* condition tend to be percent yield only, whilst the results in the *Template* condition often also include a mass or details of the purity or melting temperature.

**Fig. 4:**
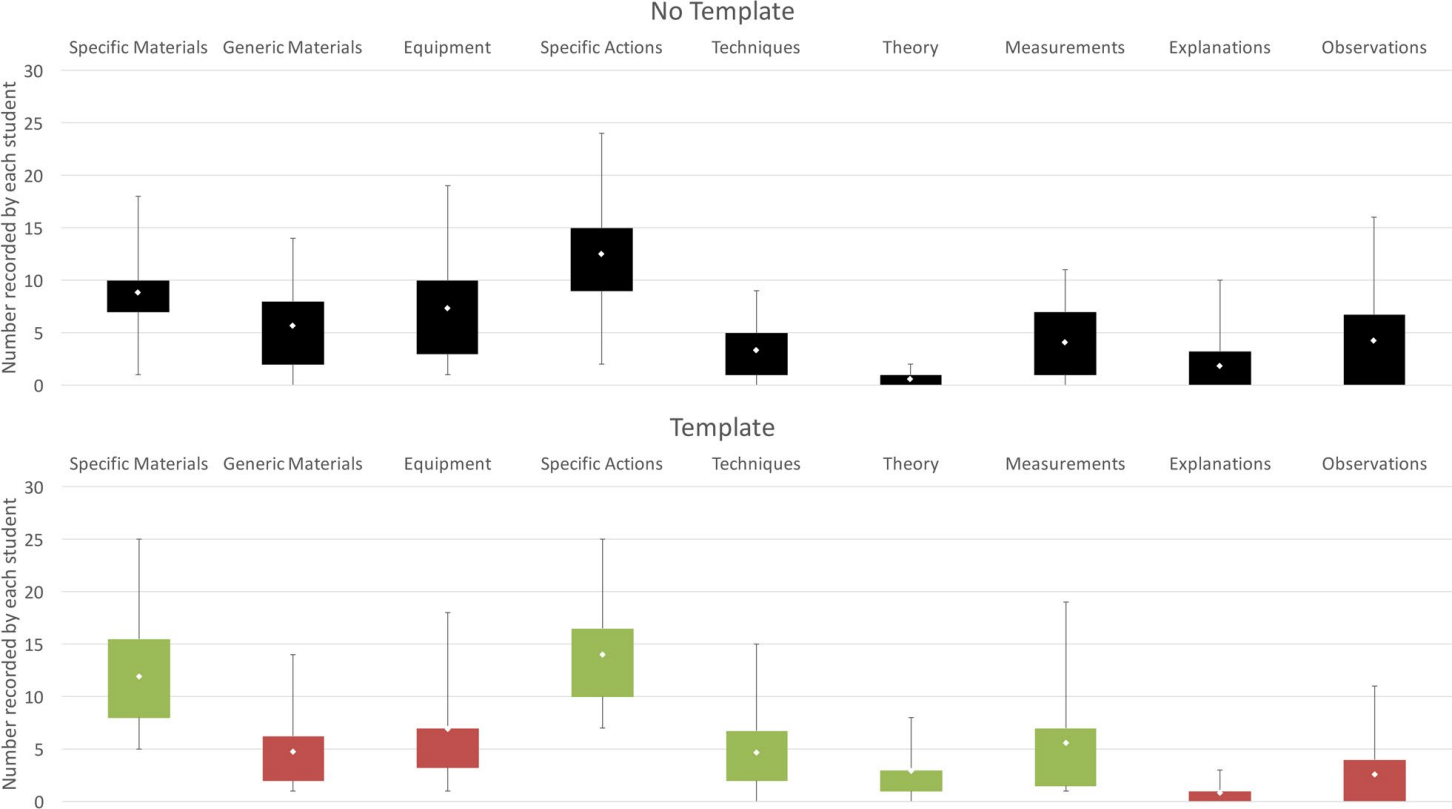
Comparison of elements recorded in the two conditions for Study 1. These *box plots* show a comparison of the range and means of other elements that were recorded in the experiment by each student (excluding Aims, Reaction Schemes, Relative Molecular Masses, and Results). The *top box plot* shows the elements that were recorded in the *No Template condition* (typically a detailed procedure of the experiment together observations and explanations for what was done). The *bottom box plot* shows the comparison for the *Template condition*. Elements *coloured in red* show a decrease in the mean and elements *coloured in green* show an increase in the mean compared to the *No Template condition*. Of particular note are the decrease in the mean for Observations (from 4.25 to 2.55) and Explanations (from 1.80 and 0.85)

Almost all of the students included materials, equipment, and actions in their report reflecting the fact that the ‘procedure’ of the experiment was the dominant information recorded in the write-ups, including observations made and explanations about particular actions, as shown in Fig. 4. The steps of the experiment, including collecting analysis information, were always recorded in the correct chronological order, even if steps were missing or the amount of detail was high or low. Although the average numbers of most of the elements recorded in each report are similar in both conditions there are some differences observed as discussed below.

**Fig. 5:**
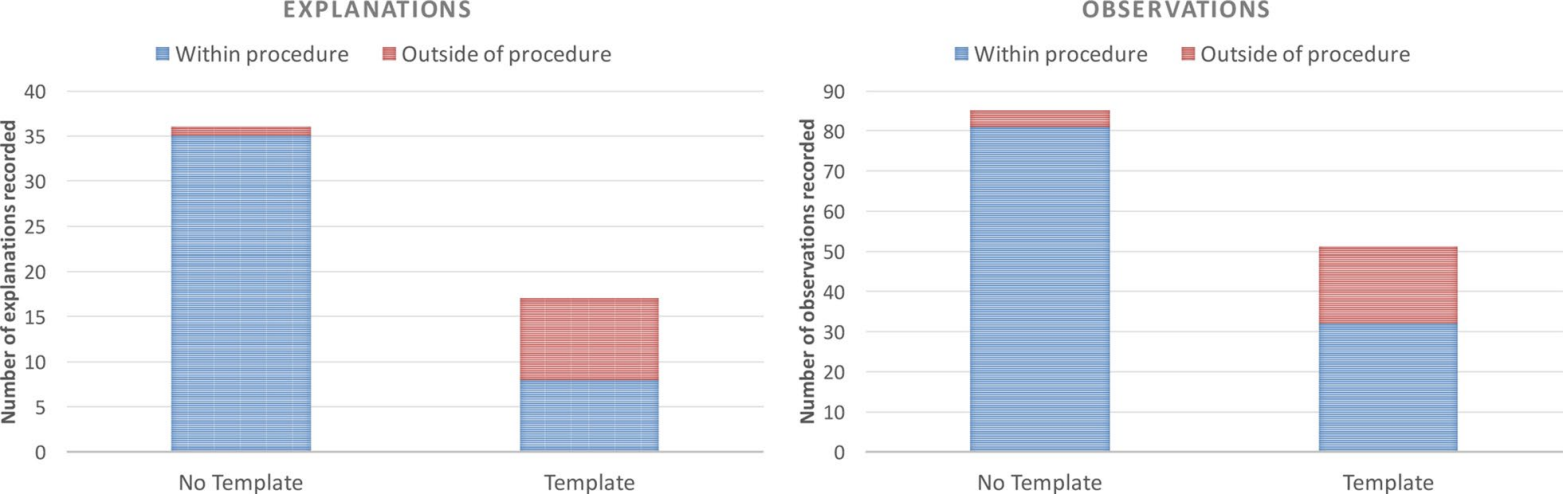
Explanations and Observations within and outside the experiment procedure in Study 1. The *bar charts* in this figure show a comparison between the total numbers of explanations and observations recorded as part of the experiment procedure and outside of the experiment procedure in each condition. As well as the overall numbers of explanations and observations being lower in the *Template condition*, they are less likely to be included as part of the description of the experiment procedure

We anticipated that using a template for recording an experiment might result in the loss of some information, possibly as a result of not asking for information specifically or a loss of personal information due to the constraints of the template. The results of this first study indicated that some types of information were more common the *No Template condition* than the *Template condition*, as shown in Fig. 4. For example, more explanations were included in the *No Template condition*, and particularly for explanations associated with actions taken in the experiment procedure, as shown in Fig. 5. Overall, a similar number of students included explanations in their reports, but the majority included explanations within their description of the experiment procedure in the *No Template condition*, compared to the *Template condition*, where many students included explanations about the experiment procedure only in the Discussion section of the template. The number of observations recorded is much higher in the *No Template condition* compared to the *Template condition*, as shown in Fig. 4, although similar numbers of students include at least one observation in both conditions. A much larger number of observations are recorded associated with their temporal occurrence in the experiment within the procedure in the *No Template condition* compared to the *Template condition*, as shown in Fig. 5. A much larger number of students included observations outside of the ‘Step-by-step’ section in the *Template condition*, together with a higher number of observations relating to their analysis of the materials created by the experiment (reflecting that more information was included about the results of the experiment in the template). In some cases observations such as the colour or state of the product are recorded in the results section of the template rather than when they occur within the procedure of the experiment. However, some personal information such as the perceived success of the experiment, learnt information, or discussions is only seen recorded in the *Template condition*; particularly within the Conclusion section for those students that completed it. The amount of ‘theory’ or ‘learned’ background information about the experiment is much higher in the *Template condition*, and more than twice as many students record this type of information compared to the *No Template* condition.

**Fig. 6:**
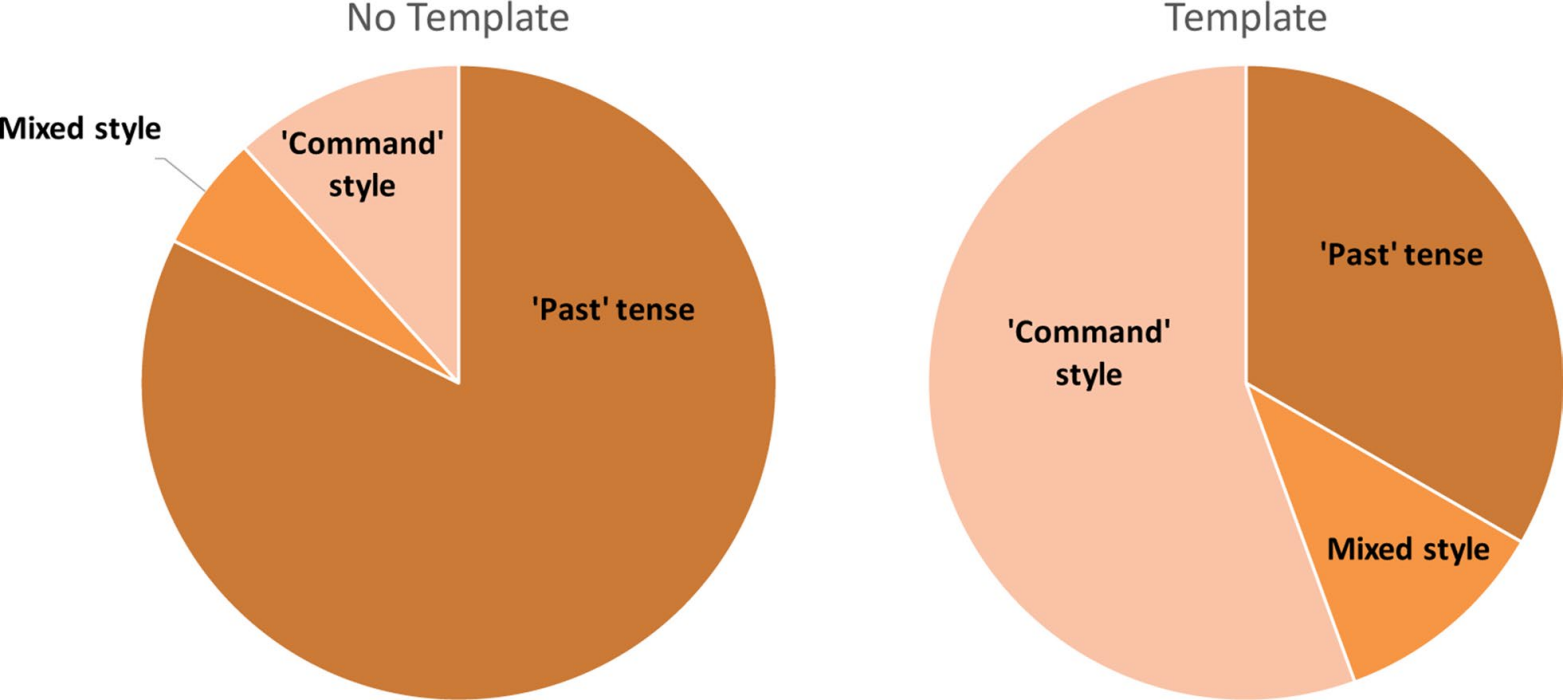
Changes in recording style observed in Study 1. The *pie charts* show changes observed in the style that the students used to record the experiment report in Study 1. In the *No Template condition* the majority of students used the ‘past tense’ to write up their experiments. In contrast, in the *Template condition* a large percentage of the students swapped style and used the imperative or command style of language

An unexpected result of the study was that a significant number of the students changed the style that they use for the report in the *Template condition*, as shown in Fig. 6. Most students use the past tense when they write about their experiment in the *No Template condition*, but half switch to using imperative sentences or a ‘command’ style in the *Template condition*. For example, “A solution of CCl_4_ and Br_2_ was poured into a dropping funnel” and “The reaction was left to cool and after another hour the TLC was taken” are sentences recorded using the past tense style, whilst “Use bunsen burner to dry flask completely” and “Add reagents, stir and bubble nitrogen through system” are sentences recorded using an imperative or command style. The dominance of use of the ‘past tense’ within the reports is unsurprising—the guidance that the students follow indicates they are expected to use the ‘third person passive past tense’ because it is considered to be objective for scientific writing.

The results of the study showed that using a template to record an experiment had both negative and positive effects, with students recording more information with the templates, but also recording less observations and explanations compared to using no template. The full results of the study can be found as Additional file 4: Data file 4.

## Study 2: OCSS and computer-based templates

A second study was undertaken to investigate whether a template that used specific questions as cues could overcome the negative effects of the template used in the first study and prompt the recording of more personal information about running an experiment, such as details of observations and decisions made, and learning as a result of completing the experiment. The new *Profile Template* included questions asking for similar information to the original template, but also some new cues, such as ‘What observations did you make in the experiment?’, ‘What did you do in the experiment?’, and ‘Did anything unexpected happen?’. The format was also changed from the paper-based questionnaires used in the first study, to computer-based questionnaires to investigate whether using a computer to capture the experiment write-up had any impact on the effects that were seen within the original study.

The format of study was the same as the first study using 20 second-year undergraduate participants from a subsequent Organic Summer School. The study included the three conditions *No Template*, *Titles Template* (template from Study 1), and the *Profile Template*. Three experiments were used in the study: preparation of an Organoboronic Acid and a Radical Benzylic Bromination from Study 1, and Carbon–Carbon Bond formation using the Suzuki Reaction, as the third experiment. The students were randomly allocated to one condition for the first experiment on 1 day and then swapped condition for their second experiment on the second day. On the third and fourth days, all students completed the Suzuki Reaction. In each condition the students were asked to complete a computer-based questionnaire independently after they had completed their experiment. All students completed the questionnaire for the *No Template condition* for their first experiment, the *Titles Template* questionnaire for their second experiment, and the *Profile Template* questionnaire for the final experiment. The responses from this study were analysed in the same way as Study 1. The questionnaires used in the study can be found as Additional file 5: Data file 5.

### Results and discussion for Study 2

One of problems of the paper-based questionnaires was that some of the students chose not to complete all sections. An advantage of the computer-based questionnaire is that the fields in the questionnaire can be made mandatory making it more likely that they will be completed. The effectiveness of the computer-based questionnaire is reflected in the results for this study with the students providing much more complete responses to the templates, with only 15 % of students missing a section from any of the templates, compared with more than half missing at least one section in the *Template condition* in the paper-based study. The students who missed one or more sections in the *Title* or *Profile* templates did not have a significant drop in word count related to these omitted sections.

**Fig. 7:**
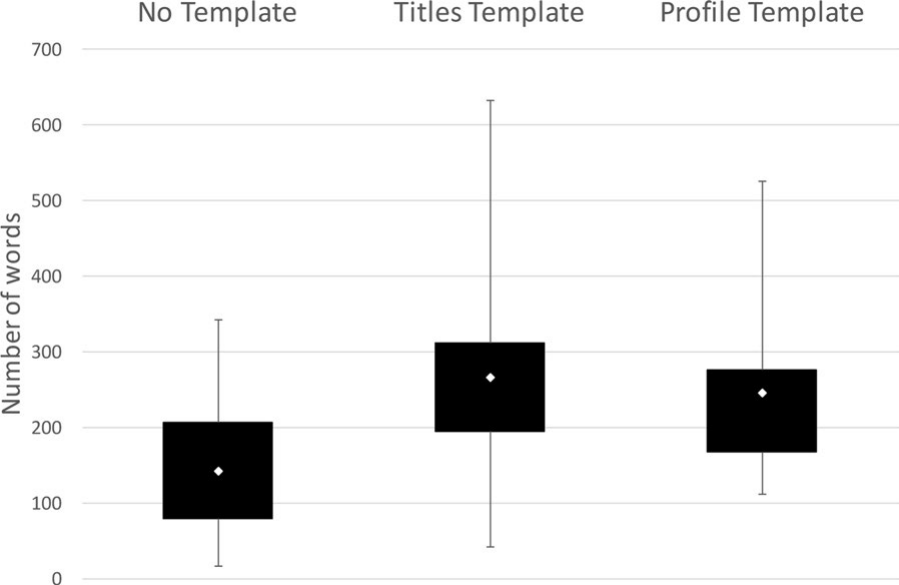
Comparison of word counts for the three conditions in Study 2. *Box plots* show the range of word counts and the means for all three conditions in Study 2. The results show that on average the number of words used in the experiment record increase for the two template conditions

In general, the average number of words increases between the *No Template* and the other template conditions, as shown in Fig. 7. These results are similar to the results seen in the first study and suggest that more information was captured in the templates compared to the *No Template condition*.

**Fig. 8:**
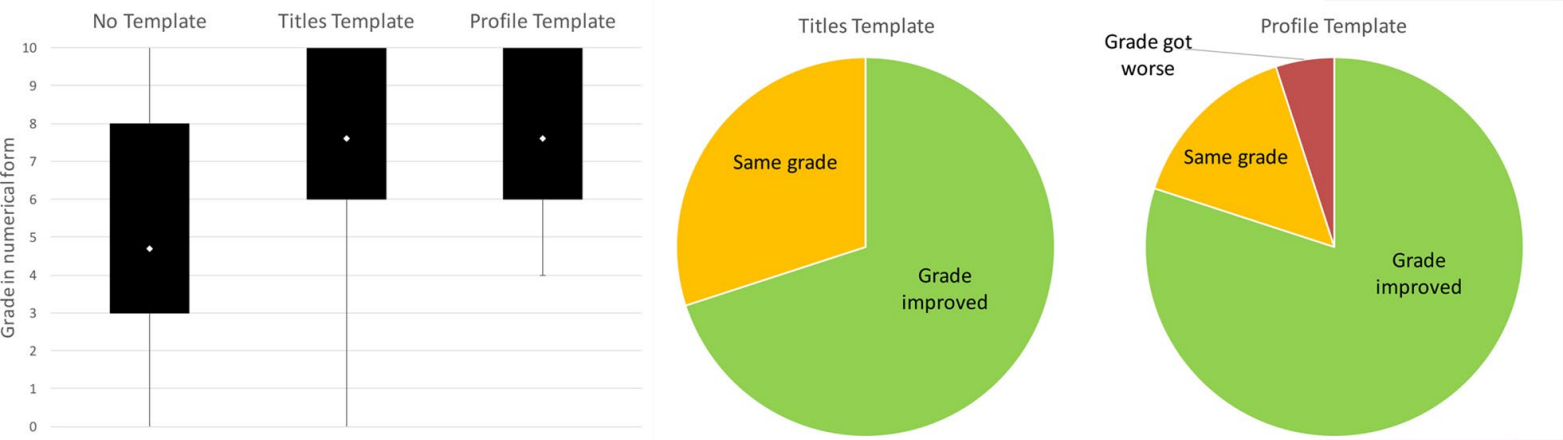
Comparison of grades for the three conditions in Study 2. *Box plots* showing the range of grades (converted into numerical form—where 0 is the lowest grade and 10 is the highest) and means for the three conditions in Study 2. The *pie charts* show how the templates affected the grades for the individual students. The figure shows that on average use of the *Titles Template* and *Profile Template* resulted in an increase in grades compared to the *No Template condition* as seen in Study 1. The mean for the two template conditions is the same, but slightly more students received an improved grade in the *Profile Template condition*

The same independent marker as used in the first study was also used to grade every report in the second study using the same quality criteria as used for Study 1. As can be seen in Fig. 8, a similar pattern is observed to the paper-based study in that both the template conditions resulted in an increase in grade compared to the *No Template condition*. Overall, slightly more students received a higher grade for the *Profile Template condition* than the *Titles Template condition.* Of the students that received the lowest grade in the *No Template condition* most improved their grade significantly in the *Titles Template condition* and all of them improved their grades significantly in the *Profile Template condition*. These results suggest that both of the templates improved the quality of the experiment record by prompting the capture of information that was otherwise forgotten.

**Fig. 9:**
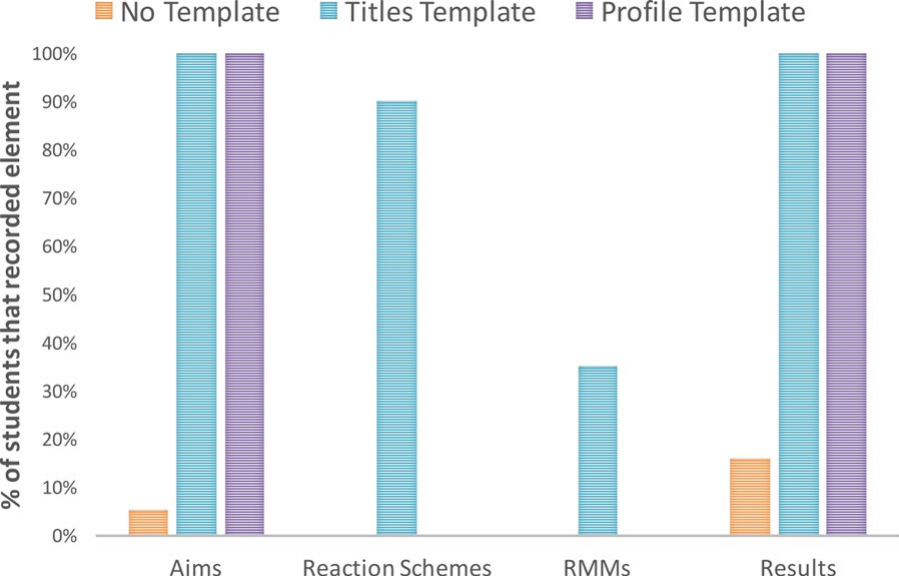
Comparison of the capture of information specifically requested in Study 2. This *bar chart* shows the percentage of students that recorded information that we specifically requested in the *Titles Template* compared to the other two conditions: Aims, Reaction Schemes, Relative Molecular Masses, and Results information. The results are similar to Study 1: all the information types are more likely to be recorded in the *Titles Template condition* where they are cued. In the *Profile Template*, only Aims and Results are specifically cued and can be seen to be recorded. It can be seen that using ‘What reactions were involved in the experiment?’ did not result in the capture of reaction scheme or RMMs in the *Profile Template condition*

In common with the first study, the template conditions do also have an effect on the topics that are recorded in the questionnaires. As shown in Fig. 9, some information is rarely or never recorded in the *No Template condition* compared, particularly compared to the *Titles Template condition* where the specific information is cued. Almost all of the students included a reaction scheme in the *Titles Template condition* (although they take different forms compared to the paper-based version), but none of the students included a reaction scheme in the *No Template* or *Profile Templates* conditions, despite the fact that the *Profile Template* provided the cue ‘What reactions were involved in the experiment?’. Instead more background theoretical information about the experiment or the reaction name were included. Only in the *Titles Template condition* were any relative molecular masses included with a similar proportion to Study 1, with one student admitting to not remembering them. In common with Study 1 more information about the results was captured in the templates, such as the actual analysis or physical appearance of the product, in addition to the product yield.

**Fig. 10:**
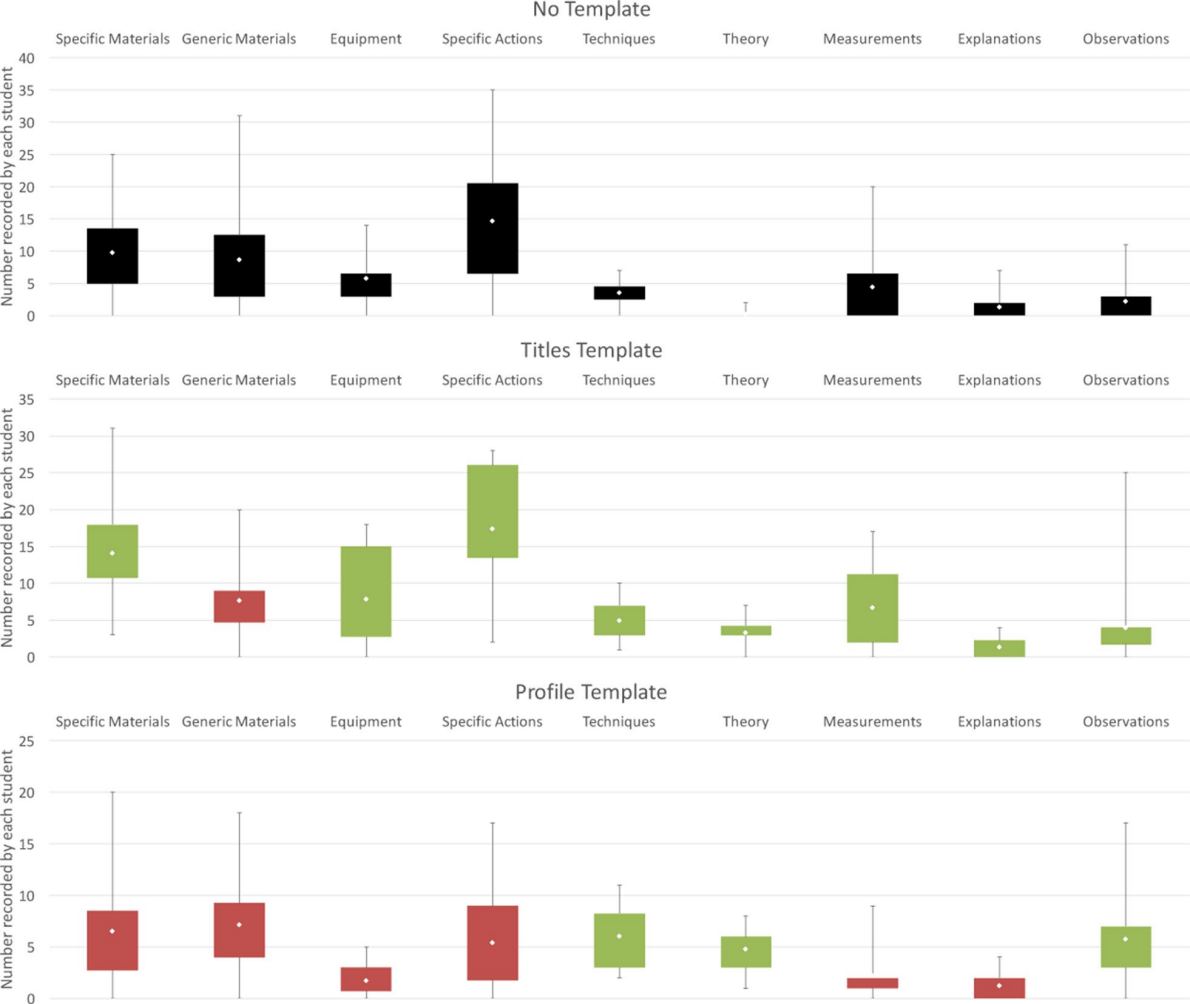
Comparison of elements recorded in the three conditions for Study 2. These *box plots* show a comparison of the range and means of other elements that were recorded in the experiment by each student (excluding Aims, Reaction Schemes, Relative Molecular Masses, and Results). The *top box plot* shows the elements that were recorded in the *No Template condition* (as before reflecting the detailed procedure of the experiment together observations and explanations for what was done). The *other box plots* show the comparison for the two template conditions, showing a difference between them in the pattern of elements recorded. As before, elements *coloured in red* show a decrease in the mean and elements *coloured in green* show an increase in the mean compared to the *No Template condition*. Of particular note are the decrease in materials, equipment, and specific actions and measurements together with a rise in the number of observations in the *Profile Template condition* reflecting the loss of detail in the experiment procedure and cue for observations in this condition

Almost all of the students included materials, equipment, and actions in their reports, again reflecting the fact that the ‘procedure’ of the experiment is the dominant information captured in the report, although the pattern of information captured is different between the two templates, as can be seen in Fig. 10. The steps of the experiment, including collecting analysis information, were always recorded in the correct chronological order, even if steps were missing or the amount of detail was high or low, as before. The average numbers of these elements included in each report is similar to those seen in the *No Template condition* and the *Titles Template condition* in Study 1.

A differences from Study 1 is seen for the *Profile Template condition*, where the average number of materials, equipment, and actions is significantly decreased in the ‘What did you do in the experiment?’ or procedural section of the template compared to the procedural information recorded in the other two conditions. A related difference is also seen with measurements recorded. In Study 1, measurements tend to be recorded as part of the experiment procedure in the *No Template condition*, and with the Results in the *Template condition*. In Study 2, a higher number of measurements are recorded within the procedure section of the *Titles Template condition* and *No Templatecondition* than in the *Profile Template condition*, reflecting the less detailed procedural information recorded in the *Profile Template condition*.

**Fig. 11:**
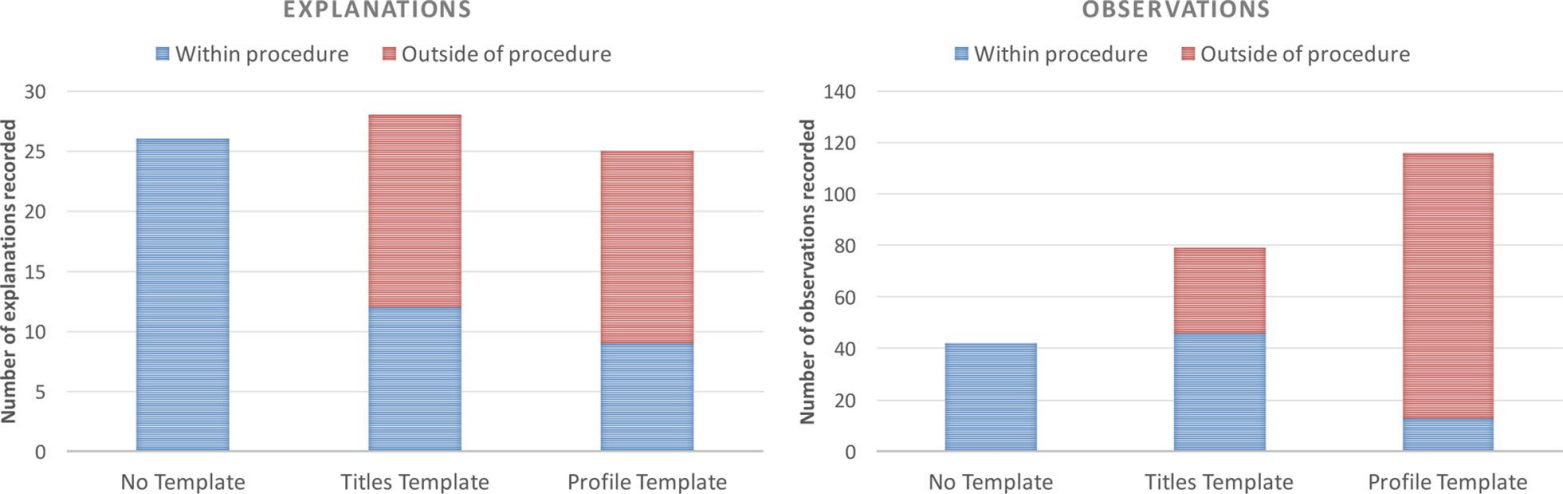
Explanations and Observations within and outside the experiment procedure in Study 2. The *bar charts* in this figure show a comparison between the total numbers of explanations and observations recorded as part of the experiment procedure and outside of the experiment procedure in each condition. In contrast to Study 1, the numbers of explanations are very similar across all three conditions, although there are significantly more included outside of the experiment procedure as before. There are a similar number of observations recorded in the *No Template* and *Titles Template condition* within the experiment procedure, but more included overall in the *Titles Template condition* with additions to the other template sections. Overall the highest number of observations are seen in the *Profile Template condition*, although very few of these are associated with the experiment procedure; the majority were included in association with the “What observations did you make in the experiment?” cue

There are some similarities and differences between Study 1 and Study 2 with the inclusion of explanations and observations in the experiment report. The average number of explanations included by each student is very similar across all the conditions, although as seen in Study 1, and can be seen in Fig. 11, fewer explanations are associated with the procedural information for the two template conditions compared to the *No Template condition*. In Study 2, the procedural information for the experiments is much more similar between the *Titles Template condition* and the *No Template condition* in Study 2. Similar numbers of observations are recorded in the ‘Step-by-step’ section of the template, with additional observations recorded in the Discussion and Conclusion sections, as shown in Fig. 11. The *Profile Template condition* has the highest number of observations, although very few of these are recorded within the procedure of the experiment; most are recorded in association with the cue ‘What observations did you make in the experiment?’ as might be expected.

The majority of ‘Conclusions’ include a ‘success statement’ about how well the experiment went, or whether the correct product was produced. In the *Titles Template condition* the Discussion and Conclusion section discuss mostly about the analysis so far, what needs to be next, and some issues around differences in results between the groups. Different groups were trying out different solvents, and some of the group had disappointment with their choice of solvent. As seen in Study 1, more learnt information and theory about the experiment was included in the template conditions, with almost all students including this kind of information in both templates, compared to only 16 % in the *No Template condition*.

For this computer-based study, no clear patterns of style change can be seen. 50 % of students start with the Past tense in the *No Template condition*, and then continue this style in both forms of the template. A change of style is seen most commonly in the *Titles Template condition*. ‘List’ is a more common style than the command style seen in the first study, although they have some similarities, with both being a briefer style of communication and including less detail. This briefer style of communication and lack of detail can be seen in from this example of the ‘list’ style covering the entire step-by-step procedure using the *Titles Template* (including spelling errors and abbreviations used by the student):

- halogenexchange reaction (THF; −78°)
- 4-buromo toluene + BuLi
- nuleophilic substitution
- B(OMe)3
- acidic work up
- 18 % HCl
- Separation
- reextract using toluene and diethylether
- The full results of the study can be found as Additional file 6: Data file 6

## Study 3: Lego Cars templates and group discussion

The final study was less formal and was carried out as part of a half-day team-building activity. The study included 60 members of Chemistry staff and research students from KTH in Stockholm. The participants were randomly allocated into 15 teams, consisting of both staff and students, and each was provided with equipment and instructions to complete an experiment using Alka-Seltzer to power Lego cars. The instructions suggested that the teams assessed the effects of using different Lego car designs and to evaluate the effects of using different quantities of Alka-Seltzer and water, thereby encouraging the teams to conduct multiple experiments. Each team was asked to record their experiments on one of three different computer-based templates randomly allocated to them. Each computer-based template was a Google word processor document, with different sections and instructions. The templates were similar to those used in Study 2, but with some key differences. The *Titles Template* included a Results table with headings customised to support the participants with the recording of their experiment results. The two template conditions also contained an additional section about the ‘Plan’ for the experiment. Again, the *No Template condition* was essentially a blank document. Some additional fields were included in each template to investigate the capture of metadata relevant to experiments, but the result of those investigations do not impact this study and are reported elsewhere [23].

This third study provided the opportunity to find out the opinions of the subjects on the different template conditions through the use of a group discussion and feedback session held after they had completed their experiments and write-ups. The questionnaires used in the study can be found as Additional file 7: Data file 7.

### Results for Study 3

In common with the first two studies, the average number of words used does vary between the different templates, although for this study the average number of words is much higher in the *Profile Template condition* compared to the *No Template condition*; the *Titles Template condition* had the lowest. One of the reasons for the low average word count in the *Titles Template condition* is that teams using this template spent most of their time completing the table and as a result recorded less information in the other sections of the template; one team completed no other sections. The presence of the table seems to have drawn attention away from the other questions and even ‘de-railed’ the experiment to some extent, with some of the teams commenting that they had less time to conduct the experiment itself because they spent so much time filling in the table. Interestingly in the *Titles Template condition* no photographs were provided, and no mentions of videos are made, in contrast to the other two conditions where the majority of teams included photographs or links to photographs in the write-ups. Several teams also mention using video to record the activity.

The majority of the write-ups in the *No Template condition* showed ‘self-structuring’, possibly because the design of the experiment encouraged running a set of repeated experiments and was also focused on producing measurements. The teams created their own structure that usually contained at least the following elements: Method; Results; Observations (measurements); Conclusions.

The write-ups for the *No Template conditions* typically contained observations, explanations, and conclusions or things that were learnt as a result of doing the experiment. The *Profile Template* generally captured more information than the other questionnaires, but the actual procedure of the experiment was recorded in less detail than in the *No Template condition*. More information was included about significant results and what was learnt as a result of the experiment in response to the specific questions about these elements. Almost all of the teams working in the *Profile Template condition* completed all of the sections in the template, in contrast to the *Titles Template condition* where many sections were not completed, as mentioned above. The inclusion of a section asking for information about what the teams planned or expected to do in the template conditions revealed differences between what they planned and what they actually did. This was particularly important information for this kind of experiment where the procedure to follow was not rigid and each team had the flexibility to perform their own version of the experiment. The results of the study can be found as Additional file 8: Data file 8.

### Study 3: Group discussion

After each of the teams had completed their experiments and write-ups, teams that had completed different versions of the templates were brought together to discuss the different templates and how their write-ups differed as a result. After their discussion each combined group verbally presented their feelings about the different templates. This activity provided an interesting opportunity to find out whether students and researchers would be comfortable using templates for recording the results of their experiments. The groups were surprised by how different their write-ups turned out when the different templates were used. Table 2 contains the comments made by the different combined teams in the group discussion.

**Table 2:** Comments from the combined teams in the group activity

Group	Comments
Red	The different template styles vary a lot. The team that used the *Profile Template condition* slavishly completed the template, but the other teams wrote notes that would help others to complete the experiment. Using a template produces a minimum level of quality and influences what is recorded. Using the *No Template condition* you can record anything you want—“free to record”—and recorded deviations and other things. The templates enable a minimum level of quality control
Blue	Using the *No Template condition* risks a lot of information being missed and therefore being unable to find it later. The *Titles Template condition* meant the team didn’t think of ideas and ideas were lost. The *Profile Template condition* asked the same questions over again, but would be best in the long term. Suggest that you can start with a template and then develop your own template over time
Green	(Only *No Template condition* and *Profile Template condition*) A lot of differences are seen between the notes that were taken. Only 10 lines were written in the *No Template condition* compared to 4 pages in the *Profile Template condition*. Most of the information is the same, but some details are missed in the *No Template condition*. It would be good to have your own template to select the information you want and be assured of getting the same information each time. Neither team checked the template before starting
Yellow	The *No Template condition* gave many degrees of freedom. More questions is equal to less freedom, but it does add different information. All the templates have their good points. More free in the *No Template condition.* A combination of all 3 is best. Good to have the guidelines to remember what to do in the experiment rather than start with an empty notebook
Orange	The team in the *Profile Template condition* didn’t read the instructions and just made the recording in a (paper-based) table and with video. The team took the approach of “capture the data first and then write up the experiment”. The team using the *No Template condition* recorded everything and managed more experiments than the others. The *Titles Template condition* team got stressed completing the table and had less time for doing the experiment. Focus on the table caused too much time to be spent on preparation. The *No Template condition* team spent some time coming up with their own structure for the experiment, which they then copied and pasted. Copying the experiment is a template in itself

The different groups raise a number of common points, in particular that they felt that the *No Template condition* gave more freedom and flexibility, but that the *Profile Template condition* led to the capture of more information, because some information could be forgotten in the *No Template condition*. Several of the groups also mention that the *Profile Template condition* structure was a good starting point for a template, but they would prefer to be able to define their own template based on their personal needs for their own experiments.

## Discussion

The guidance and training we give to students encourages them to keep consistent, clear, and accurate recordings of what they actually did in the lab and to generate reports in a standard structure. One possible outcome of the initial study in this paper was that when we handed students a blank piece of paper they would produce a write-up that would match the format that they have been trained to use. In contrast, we found the majority produced a ‘what I did’ description of the procedure, only sometimes including other information such as aims, the reaction scheme, or results. The same result is seen when the students are given a blank textbox on a computer to complete with the same instructions. These results also suggest that these students have not yet developed their own styles that more experienced researchers develop over time. Although more experienced researchers are less likely to make mistakes such as failing to record important information such as reaction schemes, deviations from the protocol, and their results, they are more likely to have biases and to use short-hand or codes for brevity, ‘local’ standards, or fail to record information that comes into the realm of ‘tacit knowledge’. Although these recording behaviours save time and mean that excessive documentation is not produced, incomplete information can make it difficult for another researcher without the same knowledge to accurately reproduce an experiment. Although many of the student reports produced in the *No Template condition* miss important information, they do often contain detailed procedures that could be repeated by another. They also include useful information about the experimenter’s personal experience, such as the observations they made, and explanations about why certain actions were taken. When the templates were used and the students changed style to record, there is a comparative loss of information with the reduction in words used, with more ‘code-like’ terms used. At the most extreme end this is seen with the ‘list’ style of recording where ‘What did you do?’ in the experiment becomes merely a list of techniques devoid of any detail, for example “reflux; workup; concentration; column purification; concentration; analysis”. These reduced steps for the procedure are similar to the short-hand ‘codes’ that some experienced researchers might use to save time. Although a lot of meaning can be derived from the steps, the original protocol is still needed to make sense of the record.

We were unsure whether providing a formal structure for the recording of experiments would be beneficial, but the use of cues to remind students and researchers to record certain information seems valuable when we note that in these studies important information is frequently missing; an observation also made by the teams from KTH in the third study. However, we were also unsure whether there might be negative consequences of using templates if they were found to constrain or otherwise change the information that is recorded in a negative way. In fact, the impact of the templates is in fact more complicated than we initially imagined. The cues provided by the templates do encourage the recording of the specific information that is prompted for, even when the prompt is simply a heading. For example, Aims are consistently recorded when subjects are asked for it in both the *Titles Template* and *Profile Template*, but are much less consistently recorded in the *No Template condition*.

Exactly what is recorded though, does depend on both the cue used in the templates, and on the experiment itself. In terms of the experiment itself, the Lego Cars experiment in Study 3 was a ‘measuring’ style of experiment, rather than a ‘making’ style of experiment and encouraged the recording of times and distances; teams recorded ‘Results’ in all of the conditions without the need for a cue. In studies one and two, the experiments involved synthetic chemistry where the aim of the experiments was to ‘make’ specific compounds. For these experiments the cues in the templates led to the recording of significantly more results than in the *No Template condition*, particularly for the computer-based Study 2, where all of the students recorded results in the templates, but only 15 % recorded them in the *No Template condition*. Differences in the nature of the protocol could also have impacted on the results recorded. The Lego Cars experiment was more explorative with only a vague protocol, whilst the synthetic chemistry experiments in Study 1 and 2 were clearly defined. More data might be expected to be recorded when the protocol is less clearly defined in order to facilitate understanding and reproducibility, whilst deviations are most important to record when the protocol is well defined. The more complete records in the Lego Cars experiment could be because each team was running an experiment of their own design, although none of the students explicitly recorded deviations from the original protocol in Study 1 and 2, possibly reflecting the relative inexperience of the undergraduate students.

The exact question or nature of the cue in the template does make a difference to the information that is recalled and recorded. This is particularly noticeable for the Reaction Schemes in the experiment write-ups. In Study 1, the presence of a cue for Reaction Scheme encourages the students to record one, although 50 % of the reports in the *No Template condition* produced reactions schemes without a cue. The same result was seen in Study 2 in the *Titles Template condition*, where all bar one of the students included a reaction scheme in some form with the report when cued. In the *Profile Template condition* the results produced for ‘What reactions were involved in the experiment?’ varied enormously, from very simple to a complex description of the underlying chemistry of the reaction, for example, “A cross-coupling reaction”, “To create a carbon carbon bond using Suzuki coupling”, “Use of the Pd catalyst in a cycle to couple the two compounds together causing the removal of PPH3 ligand”, and “Reaction of the boronic acid with the brominated benzene derivative in the presence of a palladium catalyst”. Interestingly in Study 3, the same question produces more consistent and simpler responses, for example, “HCO_3_^−^ + H^+^ = CO_2_ + H_2_O plus spectators”, “NaHCO_3_ + H_2_O → CO_2_ (g)”, “Bicarbonate in water reacts with citric acid and forms trisodium citrate and carbon dioxide.”; these differences perhaps reflect the differences in complexity between these two experiments.

In contrast to Study 1, none of the reports in the computer-based studies in the *No Template condition* recorded reaction schemes. This is most likely because capturing this information in a computer is much more difficult than on paper and the software for the study allowed text-only input. This limitation was recognised in the planning of the study, but it was also recognised as an opportunity to observe how the students handled the request to create a reaction scheme when the option to draw an image was unavailable. Although more difficult and less intuitive than drawing, the students still managed to generate a meaningful reaction scheme when asked. Many ELNs include tools to create chemical structure drawings that can be used to construct reaction schemes and other diagrams. The presence of such a tool within an ELN interface is likely to increase the creation of reactions schemes within the experiment record—partly because the tool provides a cue to do so, and partly because the tasks are potentially easier to do and can be linked to the materials recorded in the ELN. We can assume that a certain proportion of students would have included a reaction scheme within their *No Template condition* report if the software available had provided a simple mechanism to do so, based upon the findings from the *No Template condition* in Study 1. However, further investigation with tools that enable the drawing of reaction schemes would be necessary to unequivocally determine whether their presence would change the response to the cues and *No Template condition*.

Relative Molecular Masses are only recorded when the questionnaire included the cue to record it, although only about two thirds of the students recorded this information with the cue, suggesting these specific details are not well remembered and that the presence of cues alone is not enough to ensure that specific information is recorded. Many ELNs provide calculator tools that can generate RMMs and help with other calculations for the experiment. The presence of such tools will make it more likely that such information is captured in the experiment record, also helping to ensure that the record is complete and correct.

The need for more than the cue for some information is also demonstrated with the Discussion and Conclusion sections in the templates with titles. In the paper-based Study 1 a large percentage of students did not attempt to complete these sections, and many of those who did seemed to be confused, with several students describing ‘discussions’ they had during the experiment, or explanations and observations that were more relevant to the procedural section. In Study 2 the responses to these sections were mandatory, and the responses were more appropriate. The students were also able to see the questions in the computer-based questionnaire on a single page and this may have had an influence on how they responded, perhaps because the combined cues together made the traditional report structure of the sections more obvious. There is also a possibility that using the computer in this way made it easier for the students to go back and make changes to the response they had previously entered, for example if they remembered more information or wanted to refine the answer; making changes would have been more difficult and time consuming to do with the paper templates.

The original template in Study 1 did appear to have a negative effect of reducing the amount of personal information about the experiment, as predicted. Students recorded fewer explanations and observations in the *Template condition* than with free recall facilitated by the *No Template condition*. The change in style, particularly produced by the cue ‘Step by step experiment procedure’ seemed to not only reduce the number of explanations and observations recorded, but to entirely change the style of recording by many of the students, with 50 % of them changing to an imperative or ‘command’ style of wording. The change in style may suggest a change of perspective in how they were recording the information, from a ‘What I did’ style to a ‘How to do it style’, as though they were writing the instructions for a different audience. Subjects in cognitive psychology studies have been shown to recall different information when asked to recall something from a different perspective [25, 26] and it may be that different cues might prompt a different perspective to be taken when recording information. In both Study 2 and Study 3 the majority of the write-ups are produced in the past tense, although any change of style in Study 2 is still most commonly seen in the *Titles Template*. It was hoped that using the question ‘What did you do in the experiment?’ for the *Profile Template* condition would lead to the inclusion of more personal detail and explanations about what was done in the experiment, but in fact this cue resulted in a briefer description of the steps of the experiment, in some cases just a list of short phrases or ‘techniques’ describing the steps as described in the example above. The response to this question may help to explain the development of personal or established ‘codes’ to describe particular techniques by researchers. When the students in Study 2 were asked to provide a list of activities or techniques they used in the experiment, the vast majority of terms recorded were single words or short phrases; most commonly: “column chromatography”, “reflux”, “rotary evaporation”, and “TLC”. The lists also included other code-like terms such as “work up” and “purification” that contain meaning but lack specific detail; such terms may represent a ‘chunking’ of procedural steps in memory.

Details of the specific materials and the equipment that were used in the experiment were also omitted from the responses to the question. Many of the other questions in the *Profile Template condition* also often generated brief, list-like responses. The briefer answers recorded in this condition may be a result of the larger number of questions to answer or that some of the questions were more open, for example ‘What did you do in the experiment?’ is actually more open than ‘Step by step experiment procedure’. Some studies have suggested that open-ended questions may lead to a more superficial search of memory, and therefore using closed questions can generate more results [15]. This may be because the more closed questions contain more specific cues, for example ‘What chemicals or other materials did you use in the experiment?’, ‘What instruments or equipment did you use in the experiment?’, and ‘What were the aims of the experiment?’ produced sensible and specific responses from all of the students in the *Profile Template condition*. In contrast the more open question ‘What reactions were involved in the experiment?’ worked less effectively than the more specific ‘Balanced equation with relative molecular masses’, even though this was a difficult question to respond to on a computer. Another potential problem with the use of open questions may occur because chemistry students are less familiar with this style of questioning and as a consequence are not very good at constructing complete and relevant answers that reflect their actual conceptual understanding [27]. The questions asking about results, observations, what was learnt, and conclusions appeared to be more effective, perhaps because they provided a good balance between providing a sufficiently specific cue and not constraining the response, prompting the recall of information that will be useful for assessing the student’s understanding of the topic.

As mentioned by some of the teams in Study 3, there appear to be good things about all of the questionnaire styles. The difference in information that is captured in the different conditions does have an impact on the quality of the write-ups produced as reflected in the grades. In general, the use of the templates leads to higher marks than the free recall of the *No Template condition*. Although some students maintain very high or low grades throughout, a large number of students do see their grades increase significantly with the use of the template. The increase in grades is likely to be because more information about the theory, purpose, and understanding of the experiment is revealed (and recorded) in these conditions. Including the wrong kind of cue in the template can have significant negative consequences, however, constraining the information that is recorded and even potentially in influencing the way that an experiment is conducted. This was demonstrated dramatically in Study 3 where the inclusion of a ‘results table’ caused groups to fail to record other important information about the experiment, and also to spend less time performing the experiment compared to the other teams. The teams in this condition also did not record the experiment using photographs or video, unlike the majority of others who chose to record their experiments in this way. This suggests that the use of a template with a very restrictive format may also restrict creativity or change the way that recording the experiment is approached.

## Conclusions

Electronic Laboratory Notebooks have many potential advantages to academic audiences, but there are a variety of challenges to overcome. There are many advantages for ELNs to improve the quality of experiment records, for example, through the automatic linking and capture of data, flexibility to retrospectively improve the structure of notebook entries, and various built-in tools, for example, for drawing chemical structures, spell-checking, and performing calculations. These computer-based systems typically make use of forms and templates in order to capture experiment records, and a perhaps unseen challenge is the impact of these templates on the information that is captured and therefore the quality and usefulness of the experiment record. The results of the studies detailed in this paper suggest that using templates, whether paper-based questionnaires or computer-based interfaces, has both positive and negative benefits for capturing experiment records and evaluating student learning. All three of the methods for capturing information investigated in these studies have some benefits, but each also has its problems. There may be no ‘one size fits all’ template for capturing a chemistry experiment, and different cues may be more useful for different types of experiment. As suggested by the teams in Study 3, the *Profile Template* may be a good starting point for a template that can then be customised as needed for a particular user, audience, or experiment. Customisable templates are a feature of many of the major ELNs [24]. The questions that were less successful for the *Profile Template* could be improved in light of the results of these studies, to generate the recall and recording of information that is considered most important, for example, capturing student understanding of the concepts of the experiment for assessment, or specific details of a procedure or technique for future reference and reuse.

Our research has demonstrated that using templates increases the quality of the record through encouraging the recording of specific information that we requested. Using templates provides a valuable starting point acting as a reminder to researchers to record specific information. Templates could also be beneficial for researchers and organisations that need to adhere to strict guidelines on recording from funding and other stakeholder bodies; templates could be developed and even certified to meet particular standards.

However, using templates does change the information that is recorded and can result in a loss of the specific details of the procedure and the personal narrative of the experience of the experiment, critical for reproducibility and future understanding. A proposed approach to overcome the loss of the personal experience of the experiment is to make use of a ‘hybrid template’ that provides a combination of cues for recording and a formal structure, together with ‘free space’ for recording the experiment procedure and observations during the experiment.

Although more research would be needed to identify more effective combination of cues, another approach we have advocated in the past is to make use of ‘invitations’ within interfaces; invitations are prominent interface elements that encourage users to add specific content to an entry [28]. These invitations could be used instead of ‘mandatory’ fields that potentially confuse or frustrate users. Coupled with a hybrid customisable template, researchers could select to record the information that is most relevant to them, and not be ‘forced’ to record information that is not useful or confusing. Customised templates could easily be developed manually for particular disciplines, research groups, for repeating experiments, and to enable users to select whether to capture particular information in a free-form or highly structured manner. Such interfaces would need to be somewhat more sophisticated than those currently provided by ELNs to ensure maximum ease of use.

## Additional files

10.1186/s13321-016-0118-6 The template questionnaires used in Study 1.
10.1186/s13321-016-0118-6 The guidance for keeping notes provided by teaching staff to students involved in Study 1 and 2.
10.1186/s13321-016-0118-6 The guidance for writing up experiments provided by teaching staff to students involved in Study 1 and 2.
10.1186/s13321-016-0118-6 The results from Study 1.
10.1186/s13321-016-0118-6 The template questionnaires used in Study 2.
10.1186/s13321-016-0118-6 The results from Study 2.
10.1186/s13321-016-0118-6 The template questionnaires used in Study 3.
10.1186/s13321-016-0118-6 The results from Study 3.

## Abbreviation

ELN: Electronic Laboratory Notebooks

## Footnotes

1 Of 28 ELNs reviewed in 2011, only 29 % explicitly mention the use of templates [24], but of the 20 that still exist and have sufficient product information, 85 % of them use templates.
