# Supplementary material for: Effects of using structured templates for recalling chemistry experiments
Source: J Cheminform. 2016 Feb 19;8:9. doi: 10.1186/s13321-016-0118-6 (PMC4759737; doi:10.1186/s13321-016-0118-6)
Supplement: Supplementary file 1 — 10.1186/s13321-016-0118-6 The template questionnaires used in Study 1. [file 13321_2016_118_MOESM1_ESM.pdf]

ID ____ Date ________________________

Name of experiment: ____

Post-experiment write-up for Experiment x, Week x

Use the space on this piece of paper to provide a write-up of the experiment that you have just completed. Additional paper is available if you need it. Please write clearly and legibly.

ID ____ Date ________________________

Post-experiment write-up for Experiment x, Week x

Complete as fully as possible the sections in this questionnaire to provide a write-up of the experiment that you have just completed. Please write clearly and legibly. Continue on the back of the sheets if you need more space.

1. Name of experiment: ____

2. Aim of the experiment:

ID ____Date __________________________

3. Balanced equation with relative molecular masses (RMM):

ID ____Date ___________________________

4. Step by step experiment procedure:

ID ____Date ___________________________

5. Results

ID ____Date ___________________________

6. Discussion

ID ____ Date ___________________________

7. Conclusions
