# Supplementary material for: Effects of using structured templates for recalling chemistry experiments
Source: J Cheminform. 2016 Feb 19;8:9. doi: 10.1186/s13321-016-0118-6 (PMC4759737; doi:10.1186/s13321-016-0118-6)
Supplement: Supplementary file 2 — 10.1186/s13321-016-0118-6 The guidance for keeping notes provided by teaching staff to students involved in Study 1 and 2. [file 13321_2016_118_MOESM2_ESM.pdf]

Maintaining a Laboratory Notebook

One of the most useful skills to acquire in the laboratory is the proper use of a laboratory notebook. Notebooks, or other formally kept records, are an essential tool in many careers and the effort invested in developing good habits of notebook use will be amply repaid for those who pursue a future in any basic or applied sciences.

A laboratory notebook should be legible, and data in it should be readily accessible, clearly labelled with units, and grouped in a logical way. The ultimate goal of a laboratory notebook is to provide a permanent record of all the information necessary for someone else to reproduce your experiment and replicate your results.

A laboratory notebook is essentially a contemporaneous, documented, permanent, factual, and primary record of laboratory work and laboratory observations. It should be the primary source of information about the work performed in the laboratory. Everything done in the laboratory should be included in the notebook, from procedure to calculations; leaving nothing out. Even the smallest, apparently trivial, detail may make the difference.

During the experiment, you should keep a detailed account of your work, reporting everything of importance that you actually did and saw. Qualitative observations and quantitative data are best entered in a running commentary. This commentary should be recorded in the laboratory, as the experiment proceeds. It is unacceptable under all circumstances to rewrite (or “copy over”) an experiment in the notebook outside of lab. It is also unacceptable to use scrap paper for notes or to type up portions of the laboratory notebook in a word processor and then attach the printout to the notebook. Plan your activities in the laboratory so that all information is properly entered into the notebook while you are in the laboratory.

You should include all relevant data, such as the quantities of materials that you actually used (not the quantities calculated or given in the procedure, unless they are exactly the same) and the results of any analyses you performed. Raw data should be recorded with particular care; if you forget to record data at the time you measure it, or if you record it incorrectly or illegibly, the results of an entire experiment may be invalidated.

When observations are recorded in the laboratory notebook, they are always written in the passive past tense. So instead of *“I saw the solution turn green”*, write, *“The solution turned green”*. In general, personal pronouns (*e. g.* “I” & “we”) are not used in scientific writing. The use of the 3rd person passive past tense is thought to be objective. *Your* notes should not simply restate the journal, manual, or textbook procedure but should describe *in your own words* how *you* carried out the experiment.

Proper note-taking during the laboratory class is not only the necessary basis but also a tremendous help for the composition of the final report!

Important sections in a typical arrangement

Title: This should include the experiment's title, your name, the name(s) of your lab partner(s), and the date the experiment was begun.

A *balanced chemical equation* for any chemical reaction that you will be performing, including relative molecular masses for starting materials and products.

A *table of important physical properties* of all the materials (starting materials, solvents, and products) with which you will be working (be sure that you have thoroughly read the manual before preparing this table so that it includes all the chemicals that you will use). The following information must be in this table: the name of the compound, its weight or volume (with density), its relative molecular mass (if not already in the equation), and its *hazard information*. Other properties that may be important are melting points or boiling points.

Procedural Outline or Experimental Part: This section is a brief, but complete, description of the steps taken to carry out the experiment. It is not a rewrite of the source material (*e. g.* laboratory manual). All data should be recorded in chronological order. Use your own words. You may use a bulleted list for the steps. Include only procedures that you personally perform and data that you personally observe. Include all measurements made with proper units and correct number of significant figures, and any important observations noted when performing the experiment.

Yield and stoichiometry *calculations:* Based on the limiting reagent and calculating in **mol** the %-yield must be computed, taking into account significant figures and mentioning all units.

Conclusions must be included, particularly when these influenced the course of the experiment or prompted specific actions. The interpretation of the results may also be included.
