# Supplementary material for: Effects of using structured templates for recalling chemistry experiments
Source: J Cheminform. 2016 Feb 19;8:9. doi: 10.1186/s13321-016-0118-6 (PMC4759737; doi:10.1186/s13321-016-0118-6)
Supplement: Supplementary file 3 — 10.1186/s13321-016-0118-6 The guidance for writing up experiments provided by teaching staff to students involved in Study 1 and 2. [file 13321_2016_118_MOESM3_ESM.pdf]

Laboratory Report Writing

You have been supplied with a duplicate notebook for your laboratory records and spectroscopic interpretation, and a folder for your manuals and spectra. You should note that these documents are the property of the company managing your work and should be kept confidential outside of the Summer School. You also need to make sure that you keep these documents for at least two years after the completion of the OCSS as they might be called for by the company.

<u>Please note the recommendations below:</u>

1. Number the pages in the book and leave room at the front for an **index** for your experiments and their corresponding spectroscopic data. **Keep** that index **up-to-date**.
2. Complete your laboratory report in the style that you have learnt during the past years. It is essential that you complete the name, title, date, scheme, tabulation of data (molar quantity and mass/volume) and safety **before** you start the experiment.
3. A proper safety assessment will be required for your experiments. This includes the tabulation of the **hazard data** but you must include **details of the containment measures** that you anticipate needing to ensure that the risk associated with the hazard is minimised.
4. Your experimental must **describe what you actually did**, including observations, TLC results (R_F values) and analysis, and have a level of information that would allow a trained chemist to repeat your work.
5. Tabulate your spectroscopic data in full in the second half of your laboratory notebook and **keep up to date with filing** the original data. Make sure that there is an <u>unambiguous code / reference to original data</u> (see below) that links all data and reporting for the same experiment (including all individual compounds, together with side-products if you encounter any).
6. Give each experiment an unambiguous code number. Typically this will involve your initials and an appropriate number. For instance the first reaction that you do might be TAL01 and so on. In the event of getting more than one product you would use an initial integer or letter to differentiate them, *e.g.* TAL01a *et cetera*. This number should then appear on every spectrum associated with the product as well as on the sample container / vial.
7. Never use correction fluid, tippex *et cetera* in a laboratory notebook – cross out mistakes such that they remain legible and put your initials over the correction.
