# Supplementary material for: Effects of using structured templates for recalling chemistry experiments
Source: J Cheminform. 2016 Feb 19;8:9. doi: 10.1186/s13321-016-0118-6 (PMC4759737; doi:10.1186/s13321-016-0118-6)
Supplement: Supplementary file 5 — 10.1186/s13321-016-0118-6 The template questionnaires used in Study 2. [file 13321_2016_118_MOESM5_ESM.pdf]

Chemistry Summer School: Post-experiment write-up (n)

This study uses a questionnaire to collect information about the structure of knowledge and understanding that students have about an experiment that they have performed. You will complete a questionnaire to produce a brief ***post-experiment report***. There are three different questionnaires for the ***post-experiment report***, and you will complete a different questionnaire after each experiment. The questionnaires contain instructions as to how to complete them. It should take you around 20-30 minutes to complete the questionnaire.

Please do not use any notes whilst you are completing the questionnaire, this is important for ensuring that the information you record is the information that you already know or remember. The questionnaires are not an exam, if you do not know the answers to any of the questions asked it is OK to leave the question, or put that you don't know or remember. This will help us to understand whether you remember some information more than others.

We do not collect any personal or identifying information about you in this study. In any scientific reports that we prepare, we will ensure your anonymity is protected.

By clicking the button below you agree with the following statements:

- I have read these participant instructions and understand them.
- I understand that my participation is voluntary and I may withdraw at any time without my legal rights being affected.
- I understand that any personal information about me will be treated confidentially.
- I understand that the results from this study may be published and that my confidentiality will be maintained if this is the case. I consent to the published reporting of this study so long as my name or any other personal information is never used in the paper.

Many thanks for your participation in this experiment.

Section 1. About the experiment

Question 1.1

Enter your study identifier:

Question 1.2

Enter the name of the experiment you completed today:

Question 1.3

Write down up to ten things that come to mind when you think about the experiment that you completed today.

Write down these thoughts in the order that you think of them.

Section 2. Post-experiment report

Question 2.1

Use this space to provide a write-up of the experiment you have just completed.

The aim of this research was to identify what knowledge about a chemistry experiment individuals have in memory, and how the knowledge is structured. Your data will help us identify whether providing cues in the form of a template changes the knowledge that individuals recall. The results of this study will not include your name or any identifying characteristics. You may have a copy of this summary if you wish, and you may also have a copy of the research findings once the project is completed.

Thank you for your participation in this research.

Chemistry Summer School: Post-experiment write-up (t)

This study uses a questionnaire to collect information about the structure of knowledge and understanding that students have about an experiment that they have performed. You will complete a questionnaire to produce a brief ***post-experiment report***. There are three different questionnaires for the ***post-experiment report***, and you will complete a different questionnaire after each experiment. The questionnaires contain instructions as to how to complete them. It should take you around 20-30 minutes to complete the questionnaire.

Please do not use any notes whilst you are completing the questionnaire, this is important for ensuring that the information you record is the information that you already know or remember. The questionnaires are not an exam, if you do not know the answers to any of the questions asked it is OK to leave the question, or put that you don't know or remember. This will help us to understand whether you remember some information more than others.

We do not collect any personal or identifying information about you in this study. In any scientific reports that we prepare, we will ensure your anonymity is protected.

By clicking the button below you agree with the following statements:

- I have read these participant instructions and understand them.
- I understand that my participation is voluntary and I may withdraw at any time without my legal rights being affected.
- I understand that any personal information about me will be treated confidentially.
- I understand that the results from this study may be published and that my confidentiality will be maintained if this is the case. I consent to the published reporting of this study so long as my name or any other personal information is never used in the paper.

Many thanks for your participation in this experiment.

Section 1. About the experiment

Question 1.1

Enter your study identifier:

Question 1.2

Enter the name of the experiment you completed today:

Question 1.3

Write down up to ten things that come to mind when you think about the experiment that you completed today.

Write down these thoughts in the order that you think of them.

Section 2. Post-experiment report

Complete as fully as possible the questions in this section of the questionnaire to provide a write-up of the experiment that you have just completed. You can expand the size of the text boxes for each question if you need more space.

Question 2.1

Aim of the experiment:

Question 2.2

Balanced equation with relative molecular masses (RMM):

Question 2.3

Step by step experiment procedure:

Question 2.4

Results:

Question 2.5

Discussion:

Question 2.6

Conclusion:

The aim of this research was to identify what knowledge about a chemistry experiment individuals have in memory, and how the knowledge is structured. Your data will help us identify whether providing cues in the form of a template changes the knowledge that individuals recall. The results of this study will not include your name or any identifying characteristics. You may have a copy of this summary if you wish, and you may also have a copy of the research findings once the project is completed.

Thank you for your participation in this research.

Chemistry Summer School: Post-experiment write-up (p)

This study uses a questionnaire to collect information about the structure of knowledge and understanding that students have about an experiment that they have performed. You will complete a questionnaire to produce a brief ***post-experiment report***. There are three different questionnaires for the ***post-experiment report***, and you will complete a different questionnaire after each experiment. The questionnaires contain instructions as to how to complete them. It should take you around 20-30 minutes to complete the questionnaire.

Please do not use any notes whilst you are completing the questionnaire, this is important for ensuring that the information you record is the information that you already know or remember. The questionnaires are not an exam, if you do not know the answers to any of the questions asked it is OK to leave the question, or put that you don't know or remember. This will help us to understand whether you remember some information more than others.

We do not collect any personal or identifying information about you in this study. In any scientific reports that we prepare, we will ensure your anonymity is protected.

By clicking the button below you agree with the following statements:

- I have read these participant instructions and understand them.
- I understand that my participation is voluntary and I may withdraw at any time without my legal rights being affected.
- I understand that any personal information about me will be treated confidentially.
- I understand that the results from this study may be published and that my confidentiality will be maintained if this is the case. I consent to the published reporting of this study so long as my name or any other personal information is never used in the paper.

Many thanks for your participation in this experiment.

Section 1. About the experiment

Question 1.1

Enter your study identifier:

Question 1.2

Enter the name of the experiment you completed today:

Question 1.3

Write down up to ten things that come to mind when you think about the experiment that you completed today.

Write down these thoughts in the order that you think of them.

Section 2. Post-experiment report

Complete as fully as possible the sections in this questionnaire to provide a write-up of the experiment that you have just completed.

Question 2.1

What were the aims of the experiment?

Question 2.2

What reactions were involved in the experiment?

Question 2.3

What did you do in the experiment?

Question 2.4

What observations did you make in the experiment?

Question 2.5

What were your results?

Question 2.6

Did anything unexpected happen?

Question 2.7

What did you learn from the experiment?

Question 2.8

What are your conclusions about the experiment?

Section 3. Experiment profile

Question 3.1

What chemicals or other materials did you use in the experiment?

Question 3.2

What instruments or equipment did you use in the experiment?

Question 3.3

Where did you do the experiment?

Question 3.4

What activities or techniques did you use in the experiment?

Question 3.5

What other information might be useful to remember about the experiment?

For example: sample identifiers, safety information, settings for the analysis?

The aim of this research was to identify what knowledge about a chemistry experiment individuals have in memory, and how the knowledge is structured. Your data will help us identify whether providing cues in the form of a template changes the knowledge that individuals recall. The results of this study will not include your name or any identifying characteristics. You may have a copy of this summary if you wish, and you may also have a copy of the research findings once the project is completed.

Thank you for your participation in this research.
