# Supplementary material for: Effects of using structured templates for recalling chemistry experiments
Source: J Cheminform. 2016 Feb 19;8:9. doi: 10.1186/s13321-016-0118-6 (PMC4759737; doi:10.1186/s13321-016-0118-6)
Supplement: Supplementary file 7 — 10.1186/s13321-016-0118-6 The template questionnaires used in Study 3. [file 13321_2016_118_MOESM7_ESM.pdf]

LEGO ROCKET CARS

Write down as many words or short phrases as you can that come to mind when you think about the experiment:

Record your experiment below:

LEGO ROCKET CARS

Record the details of your experiment in the sections below.

Title:

Keywords:

Objective:

Plan:

Reaction:

Procedure:

Results:

Run Number ($\frac{1}{4}$ tablet)	Quantity of water ($\frac{1}{2}$, $\frac{1}{3}$, $\frac{1}{4}$)	Weight of car (g)	Aerodynamic car shape (Yes / No)	Time to launch (seconds)	Distance travelled (cm)	Comments
1						
2						
3						
4						
5						

6						
7						
8						

Conclusions:

LEGO ROCKET CARS

What are the aims of the experiment?:

What reactions are involved in the experiment?

What do you plan to do in the experiment?

What did you actually do in the experiment?

What observations did you make in the experiment?

What were your results?

Did anything unexpected happen?

What did you learn from the experiment?

What are your conclusions about the experiment?

Who took part in the experiment?

What chemicals or other materials did you use in the experiment?

What instruments or equipment did you use in the experiment?

Where did you do the experiment?

What activities or techniques did you use in the experiment?

What other information might be useful to remember about the experiment?
